# Murine Cell Line Models for Vascular Mimicry: The Role of YAP/TAZ Signaling

**DOI:** 10.3390/ijms26189129

**Published:** 2025-09-18

**Authors:** Matilde Righetti, Ana-Maria Primorac, Janine Terra Erler, Victor Oginga Oria

**Affiliations:** Biotech Research and Innovation Center (BRIC), Faculty of Health and Medical Sciences, University of Copenhagen, Ole Maaløes Vej 5, DK-2200 Copenhagen, Denmark; matilde.righetti@bric.ku.dk (M.R.); srd896@alumni.ku.dk (A.-M.P.)

**Keywords:** vascular mimicry, syngeneic mouse models, CT26, KPC, YAP/TAZ

## Abstract

Vascular mimicry (VM) refers to the formation of vessel-like structures by tumor cells independent of endothelial cells. These VM channels connect to the host’s vascular network and are associated with aggressive tumors and poor patient prognosis. Most VM research has been conducted on melanoma, relying on patient-derived and mouse cell lines. In other solid tumors, VM studies rely on human cell lines, which have certain limitations for in vivo studies. Specifically, most in vivo VM research involving human cells uses subcutaneous mouse models that fail to recapitulate organ-specific tumor microenvironments. As the microenvironment is an essential driver of tumor vascularization, including VM, murine cell lines could facilitate VM investigations in syngeneic mouse models. Here, we present CT26 and KPC, well-characterized murine colorectal and pancreatic cancer cell lines, as cell models for VM investigations. Using in vitro cell-based assays, we demonstrate that CT26 and KPC undergo VM, a cell-intrinsic process that is enhanced by serum deprivation and exposure to hypoxia and is independent of tumor-secreted growth factors. Additionally, we demonstrate the importance of YAP/TAZ signaling in VM formation, as inhibition at non-cytotoxic concentrations attenuated VM formation. Remarkably, CA3, the most potent of the two inhibitors, significantly reduced cell proliferation in both cell lines at the IC_50_ concentration. This reduction in cell proliferation was associated with the induction of apoptosis in CT26 cells and changes in the cell cycle in both CT26 and KPC cells. Finally, dual YAP/TAZ knockdown in both cell lines significantly abrogated VM formation, validating our initial findings using inhibitors. These results show that CT26 and KPC cells undergo VM, and given their extensive use in cancer research, can be used to investigate VM in vivo using syngeneic models.

## 1. Introduction

One of the accepted principles underlying tumor survival is the constant supply of nutrients and oxygen to sustain malignant growth and metastasis. This is why tumor angiogenesis was identified as one of the hallmarks of cancer and later targeted with anti-angiogenic therapies [1,2]. However, most anti-angiogenic therapies have failed in the clinic [1,3], and these failures have provided novel insights into other forms of vasculature that perfuse tumors other than angiogenesis. It is now evident that tumors can develop alternative vascularization mechanisms such as mosaic vessels and vascular mimicry (VM) [4,5]. VM, first described in uveal and metastatic cutaneous melanomas [2,4,5], is a functional and intricate network of vessel-like structures formed by aggressive tumor cells instead of endothelial cells, and may contribute to the failure of anti-angiogenic therapies [3]. As an alternative to angiogenic networks, VM tubules supply sufficient nutrients and oxygen to rapidly growing tumors. VM is a complex, multistep process that involves the activation and proliferation of tumor cells and differentiation into endothelial-like cells, followed by the formation of vessel-like networks. Notably, VM and other alternative vascularization mechanisms have been demonstrated to promote tumor initiation, growth, distant metastasis, and poor prognosis of multiple cancer types [6]. While VM has been extensively studied in primary melanomas [5,7] and metastatic melanoma [8,9], it also occurs in many other cancer types, including renal cell carcinoma [10], glioblastoma [11], ovarian cancer [12], colorectal cancer [13], pancreatic cancer [14], lung cancer [15], and breast cancer [16].

Many published studies involving VM, rightly, rely on human cell lines to model VM both in vitro and in vivo. Even in human melanoma, where VM has been studied extensively, modeling this phenomenon relies on either established human melanoma cell lines or short-term patient-derived primary cultures [5,9]. While it is essential to demonstrate VM formation using these human cancer cells, several limitations are associated with them in the context of drug discovery. Syngeneic mouse models mimic the clinical tumor’s microenvironment and are important for target discovery and validation [17]. Yet, in the context of VM, as most studies rely on human cell lines, in vivo studies have been limited to subcutaneous implantations, which lack aspects of the microenvironment and offer limited information beyond tumor growth [17,18]. Interestingly, a few VM studies on melanoma have incorporated the murine cell lines B16F10, YUMM1.7, and YUMMER1.7, which undergo VM in vitro and in vivo [8,9,19], allowing for the study of spontaneous orthotopic and experimental metastasis in immunocompetent syngeneic models. As VM is associated with aggressive disease, distant metastasis, and resistance to current treatments, understanding the molecular mechanisms is vital for the development of future therapies. This is especially true for malignancies such as pancreatic ductal adenocarcinoma (PDAC) and metastatic colorectal cancer (mCRC), which are hypovascular and unresponsive to anti-angiogenic therapies, yet highly aggressive with poor outcomes [20,21,22]. A recent study demonstrated that VM formation in PDAC is strongly linked to its hypovascular and desmoplastic microenvironment, the latter of which limits endothelial cell-driven angiogenesis [23]. The available studies focusing on VM in both PDAC and mCRC utilize human cancer cell lines, which provide valuable insights into the molecular mechanisms of VM. However, in vivo, most of these studies employ subcutaneous models or orthotopic xenografts in immunocompromised mice to investigate the effects of the identified pathways or VM inhibitors, with tumor growth or volume as the primary outcome [24,25,26]. This limits our understanding of VM signaling processes in vivo and necessitates the investigation of VM in established murine cell lines that can be utilized in immunocompetent mouse models.

In this study, we characterized VM formation in CT26 and KPC cells, murine cell lines for CRC and PDAC, respectively. We demonstrate their suitability as models for VM formation in vitro and identify the triggers of VM in these cell lines. Additionally, we hypothesized that VM is a cell-intrinsic mechanism regulated by YAP/TAZ signaling, thus representing suitable models for syngeneic in vivo studies.

## 2. Results

### 2.1. Vascular Mimicry Is Dependent on Serum Starvation

**Figure 1:**
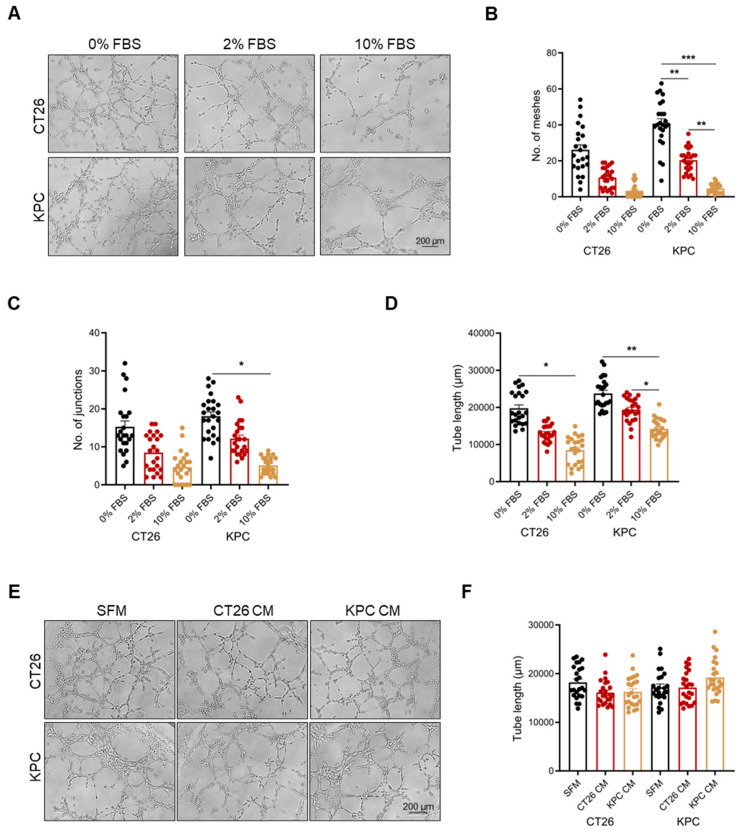
Vascular mimicry is dependent on serum deprivation. (**A**) Images of CT26 and KPC cell VM formation. Quantification of the number of (**B**) meshes, (**C**) junctions and (**D**) tube length in CT26 and KPC cells undergoing VM in the presence of increasing levels of FBS (*n* = 8 images per independent biological experiment). (**E**) Images of CT26 and KPC cell VM formation in the presence of tumor-conditioned medium. (**F**) Quantification of the total tube length in CT26 and KPC cells in the presence of serum-free growth medium and tumor-conditioned medium (*n* = 8 images per independent biological experiment). All data are expressed as mean ± SEM of three independent experiments, and statistical significance is determined Welch’s ANOVA test paired with Dunnett’s T3 multiple comparisons test: * *p* < 0.05, ** *p* < 0.01, and *** *p* < 0.001.

VM formation in CT26 and KPC cells was assessed by their capacity to form vascular networks when cultured on Matrigel in vitro. Our results showed that CT26 and KPC cells were able to form tube-like structures (Figure 1A). Intriguingly, just like in endothelial- cell-driven angiogenesis, VM formation in both cell lines was dependent on serum deprivation, as demonstrated by the decreased tube length, as well as the reduced number of meshes and junctions, with increasing concentrations of FBS (Figure 1B–D).

### 2.2. Vascular Mimicry Is a Cell-Intrinsic Mechanism Independent of Tumor-Secreted Factors

Next, we evaluated whether this vascularization process is dependent on secreted factors. This is because pathological angiogenesis, the most dominant form of tumor vascularization, is mainly dependent on secreted factors such as vascular endothelial growth factor (VEGFA) and angiopoietins [1]. As an initial step, we used conditioned medium from CT26 or KPC on 4T1 cells, a non-VM-forming murine cell line (Supplementary Figure S1A), to determine whether we could induce VM formation. Using either CT26- or KPC-conditioned medium, 4T1 cells remained unable to form these vessel-like structures (Supplementary Figure S1B). To validate these observations, we investigated whether we could enhance VM formation in CT26 or KPC cells by using tumor-conditioned medium from either cell line. We reasoned that if VM formation was dependent on secreted factors, we could enhance VM formation in the presence of tumor-conditioned medium. We did not observe any increase in VM formation in either cell line in the presence of tumor-conditioned medium (Figure 1E,F), reiterating that VM formation is a cell-intrinsic mechanism.

### 2.3. Hypoxia-Educated Tumor Cells Enhance Vascular Mimicry Formation

**Figure 2:**
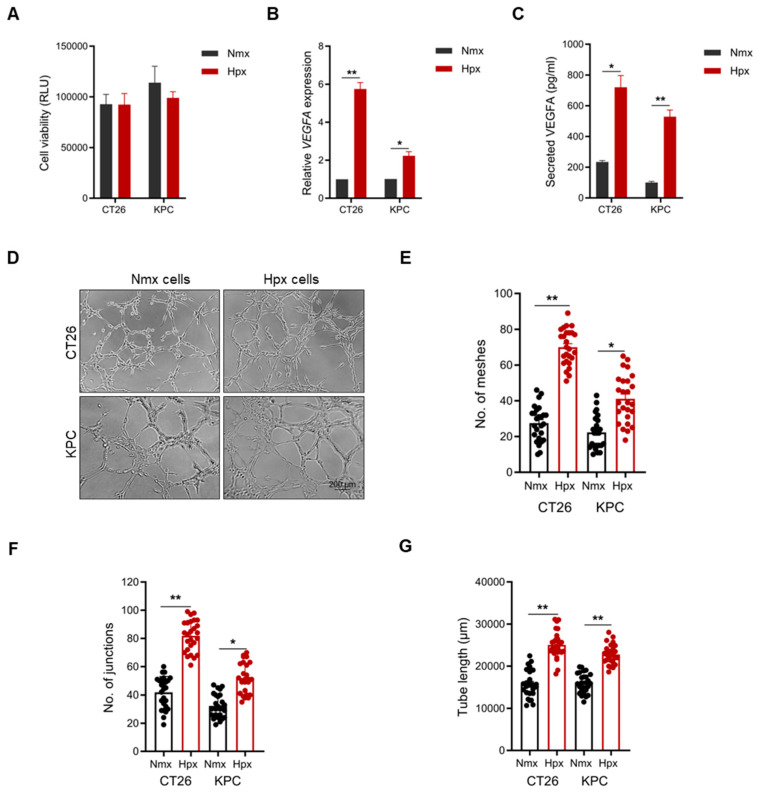
Effect of hypoxia on vascular mimicry formation. (**A**) Cell viability of CT26 and KPC cells cultured under normoxic or hypoxic conditions for 24 h. (**B**) mRNA gene expression of VEGFA and (**C**) secreted VEGFA protein levels in CT26 and KPC cells after culture in normoxic or hypoxic conditions for 24 h. (**D**) Images of normoxia- and hypoxia-educated CT26 and KPC cells undergoing VM formation. Quantification of the number of (**E**) meshes and (**F**) junctions and (**G**) tube length in normoxia- and hypoxia-educated CT26 and KPC cells undergoing VM (*n* = 8 images per independent biological experiment). All data are expressed as mean ± SEM of three independent biological experiments, and statistical significance is determined using unpaired two-tailed *t*-test with Welch’s correction: * *p* < 0.05 and ** *p* < 0.01.

Accumulating evidence has demonstrated the significance of hypoxia in enhancing VM in aggressive solid tumors [25,27]. To this end, we tested whether hypoxic tumor-conditioned medium would enhance VM formation in either cell line. First, we demonstrated the presence of hypoxia by quantifying the levels of VEGFA mRNA by qPCR and secreted protein by ELISA, without affecting cell viability (Figure 2A–C). Next, we evaluated whether hypoxic tumor-conditioned medium would enhance VM formation in CT26 or KPC cells cultured under standard normoxic conditions. Our results showed that hypoxic tumor-conditioned medium did not enhance VM formation in either CT26 or KPC cells (Supplementary Figure S1C–F).

As our findings pointed toward a cell-intrinsic mechanism, we then evaluated whether cells cultured under hypoxia and subsequently used in VM assays would enhance vascular mimicry formation. Our data showed that cells cultured under hypoxia exhibited significantly elevated VM formation compared to their normoxic counterparts (Figure 2D). For both cell lines, there was a significant increase in tube length, as well as the number of meshes and junctions, in hypoxia-educated cells compared to normoxic cells (Figure 2E–G). These findings strengthen our earlier observations that VM is a cell-intrinsic mechanism rather than being mediated by paracrine signaling through secreted factors.

### 2.4. YAP/TAZ Inhibition Significantly Reduces Vascular Mimicry

**Figure 3:**
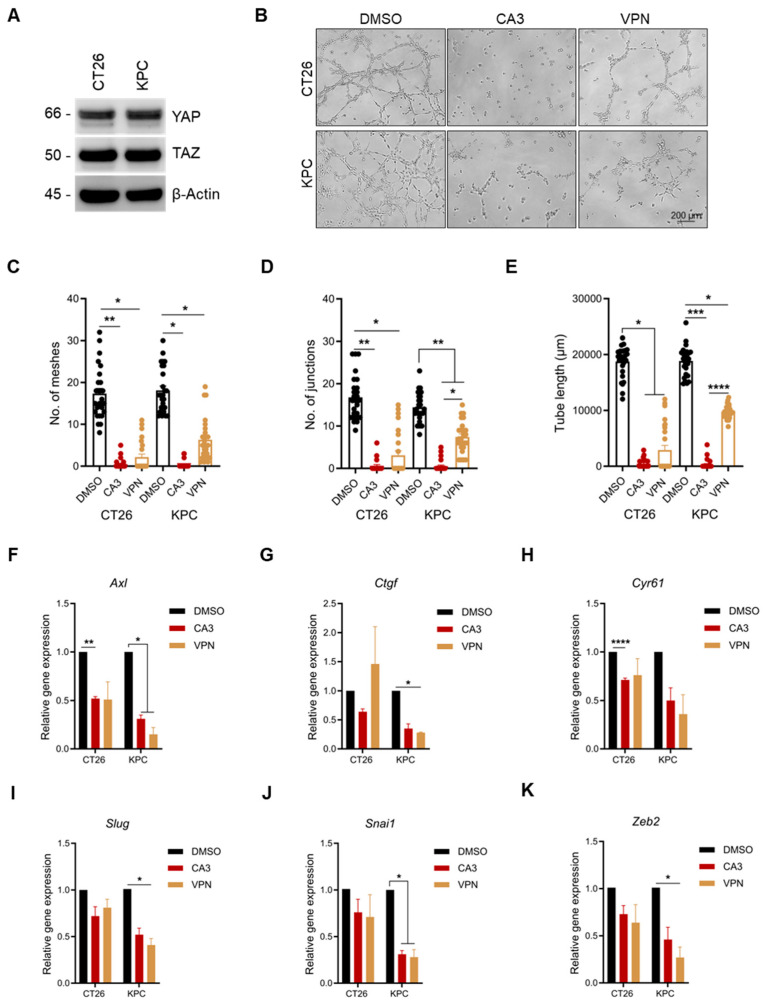
YAP/TAZ inhibition reduces vascular mimicry formation. (**A**) Immunoblot analysis of YAP and TAZ in CT26 and KPC. (**B**) Images of CT26 and KPC cell VM formation in the presence of either 500 nM of CA3 or Verteporfin. Quantification of the number of (**C**) meshes and (**D**) junctions and (**E**) tube length in CT26 and KPC cells treated with either CA3 or Verteporfin (*n* = 8 images per independent biological experiment). Relative gene expression analysis of YAP/TAZ transcriptional targets (**F**) Axl, (**G**) *Ctgf* (*n* = 2 for KPC cells), (**H**) *Cyr61*, (**I**) *Slug*, (**J**) *Snai1*, and (**K**) *Zeb2* in CT26 and KPC cells following treatment with either CA3 or Verteporfin. All data are expressed as mean ± SEM of three independent experiments, and statistical significance is determined Welch’s ANOVA test paired with Dunnett’s T3 multiple comparisons test: * *p* < 0.05, ** *p* < 0.01, *** *p* < 0.001, and **** *p* < 0.0001.

The Hippo pathway has been previously implicated in VM formation in many solid tumors, including melanoma, glioblastoma, lung, colorectal, and pancreatic cancers [2,9,27]. As our cell models, CT26 and KPC, undergo VM, we confirmed the expression of YAP and TAZ, the functional effectors of the Hippo pathway, via Western blotting (Figure 3A). Next, we investigated whether the pharmacological inhibition of YAP, using two known inhibitors, CA3 and Verteporfin, affected VM formation in these cell lines. We investigated a drug concentration that does not induce cytotoxicity in these cells, such that any differences observed in VM formation are not due to cytotoxicity. As VM formation was analyzed 6 h post-culture, we assessed the viability of CT26 and KPC cells in the presence of either inhibitor. Our results showed that at 500 nM of either inhibitor, there were no differences in cell viability compared to our control treatment (Supplementary Figure S2A), corroborating findings from our previous study [9]. We then evaluated the effect of these inhibitors on VM formation. In both cell lines, there was a significant decrease in VM formation in the treated groups, as demonstrated by the reduced number of meshes and junctions and reduced tube length (Figure 3B–E). Interestingly, CA3 had a more substantial inhibitory effect on KPC cells than Verteporfin. We concluded that the differences observed between our control and treatment groups are solely linked to YAP inhibition and not a consequence of cell cytotoxicity. To gain a deeper understanding of how the two inhibitors affect VM formation, we examined the gene expression levels of known YAP/TAZ target genes. We relied on our previous findings, where we demonstrated that YAP inhibition by either drug led to cell-dependent decreased expression in multiple YAP/TAZ gene targets after 6 h [9]. At 6 h, there were no changes in the expression of the YAP/TAZ gene targets. However, we observed a decrease in select YAP/TAZ target genes in both cell lines after 24 h (Figure 3F–K, Supplementary Figure S2B).

### 2.5. VM-Inhibiting Dose Attenuates Tumor Cell Chemotaxis

Using the same non-cytotoxic VM-inhibiting dose, we evaluated the effects of CA3 and Verteporfin on the proliferation, colony formation, and chemotaxis of CT26 and KPC cells in vitro. Using the CellCyte X Live Cell Imaging system, we found that the VM-inhibiting dose did not affect the proliferation of CT26 or KPC cells (Supplementary Figure S2C–D). Next, we investigated the colony formation capacity of our cell lines in the presence of these inhibitors. As CT26s grow as individual cells and are unable to establish colonies, this assay was conducted with KPC cells only. Interestingly, while the VM-inhibiting CA3 dose also inhibited colony formation, the Verteporfin dose had no effect on colony formation (Supplementary Figure S2E,F). Chemotaxis within the TME plays an important role in neovascularization, including angiogenesis and vascular mimicry [2,28]. We evaluated the effect of a VM-inhibiting dose for either inhibitor on CT26 and KPC chemotaxis, using 10% FBS as a chemoattractant. YAP/TAZ inhibition led to a reduction in tumor chemotaxis in the presence of either inhibitor, with statistical significance in CT26 cell line (Supplementary Figure S2G,H). Taken together, these results suggest that VM formation is, in part, dependent on chemotactic migration.

### 2.6. Determination of IC50 Concentration and Subsequent Functional Effect on Cancer Cells

**Figure 4:**
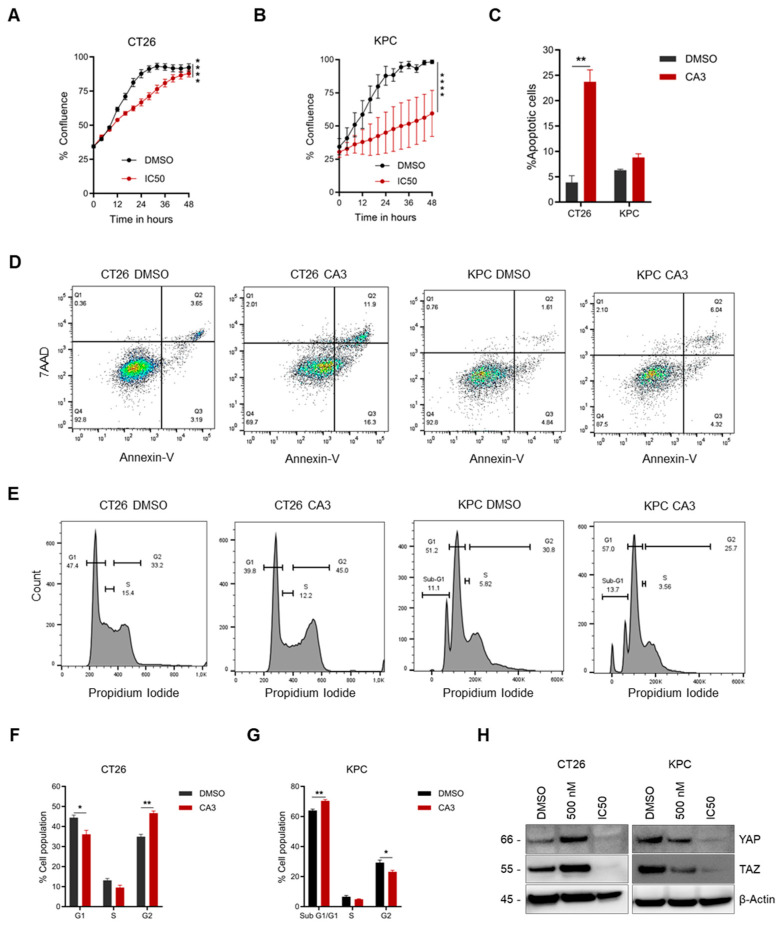
Functional characterization of CA3-mediated YAP/TAZ inhibition. (**A**,**B**) The proliferation curves of CT26 and KPC cultured in the presence of CA3 at an IC_50_ concentration. Growth curve differences were calculated using area under the curve (AUC). (**C**) CA3-mediated apoptosis in CT26 and KPC cells. Cells were treated with CA3 (IC_50_ concentration) for 24 h, and apoptosis was evaluated by Annexin V/7AAD staining and flow cytometry analysis. (**D**) Flow cytometry scatter plots of CA3-treated CT26 and KPC cells. (**E**) Distribution of cell cycle phases in CT26 and KPC cells treated with CA3 for 24 h, stained with propidium iodide, and analyzed by flow cytometry. (**F**,**G**) The percentage of cells in each phase of the cell cycle in CT26 and KPC cells, respectively, is shown in E. (**H**) Immunoblot analysis of YAP and TAZ in CT26 and KPC cells after treatment with CA3. All data are expressed as mean ± SEM of three independent biological experiments, and statistical significance is determined using unpaired two-tailed *t*-test with Welch’s correction: * *p* < 0.05, ** *p* < 0.01, and **** *p* < 0.0001.

Given our findings using the VM-inhibiting dose of either drug, we were curious to determine whether the IC_50_ concentrations of either drug would inhibit additional functional processes such as proliferation and invasion. To this end, the IC_50_ of either drug was evaluated using the Cell Titer Glo^TM^ Cell Viability Assay. As the IC_50_ for Verteporfin was too high (>10 µM), CA3 was used for subsequent functional assays, as its IC_50_ values fell within the clinically relevant range (Supplementary Figure S2I,J). First, the effect of CA3 on cell proliferation was investigated using the IncuCyte X^TM^ Live Cell Imager. Our results showed that CA3 (IC_50_ value) significantly inhibited proliferation in both cell lines (Figure 4A,B). We hypothesized that the observed CA3-mediated decrease in cell proliferation may be due to the induction of apoptosis or changes in cell cycle progression, as previously reported [29,30]. Treatment of either cell line with its respective CA3 IC_50_ concentration led to a cell-dependent apoptotic effect. While CT26 cells were sensitive to CA3, which significantly increased apoptosis compared to the control, KPC cells were insensitive to CA3 treatment (Figure 4C,D). Additionally, we also observed the cell-dependent effects of CA3 on cell cycle progression. While treating CT26 cells with CA3 led to an increase in cells in the G2 phase, in KPC cells, there was a marked increase in cells in the sub-G1/G1 phase post-treatment with CA3 (Figure 4E–G). These observations could be partly associated with the loss of YAP/TAZ protein expression following treatment with CA3 at an IC_50_ concentration (Figure 4H).

### 2.7. Dual YAP/TAZ Gene Knockdown Attenuates VM Formation

**Figure 5:**
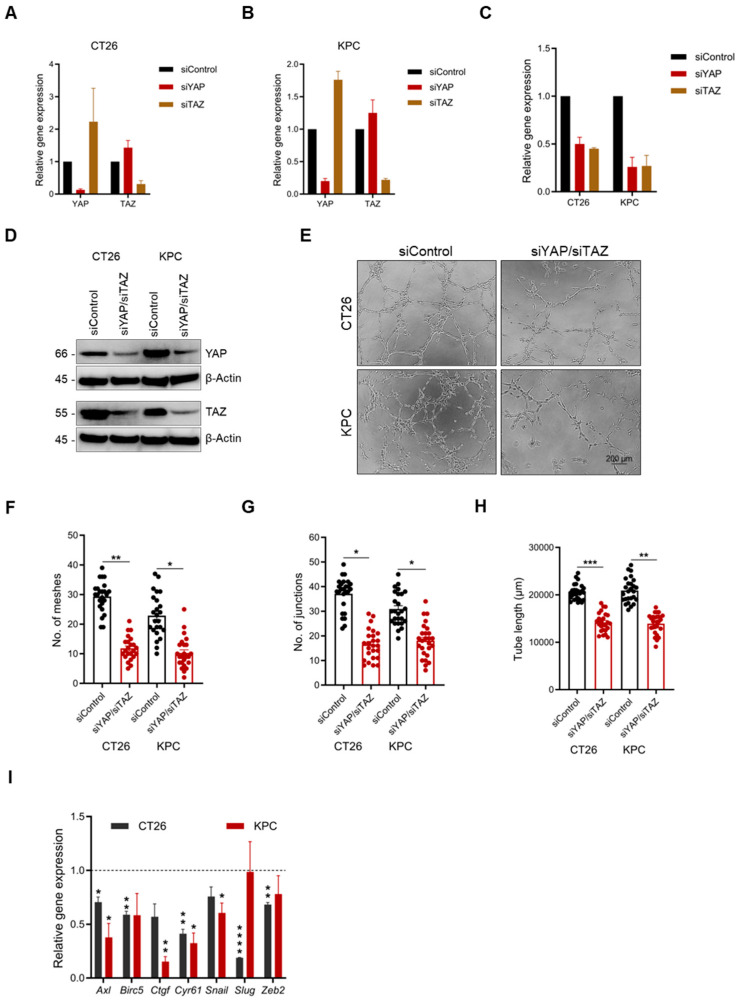
YAP/TAZ knockdown attenuates vascular mimicry. (**A**,**B**) Expression silencing of YAP and TAZ genes in CT26 and KPC cells using siRNAs. Dual gene knockdown of *YAP*/*TAZ* in CT26 (*n* = 2 for siTAZ) and KPC cells evaluated by (**C**) qPCR and (**D**) immunoblotting. (**E**) Images of CT26 and KPC cell (siControl vs. siYAP/siTAZ) VM formation. Quantification of the number of (**F**) meshes and (**G**) junctions and (**H**) tube length in CT26 and KPC cells following dual *YAP*/*TAZ* gene knockdown (*n* = 8 images per independent biological experiment). (**I**) Relative gene expression analysis of *YAP*/*TAZ* transcriptional targets in CT26 and KPC cells following dual *YAP*/*TAZ* knockdown. All data are expressed as mean ± SEM of three independent biological experiments, and statistical significance is determined using unpaired two-tailed *t*-test with Welch’s correction: * *p* < 0.05, ** *p* < 0.01, *** *p* < 0.001, and **** *p* < 0.0001.

Pharmacological inhibition of YAP/TAZ-mediated VM formation was corroborated by YAP/TAZ knockdown. Initially, YAP and TAZ gene expression post-transfection was evaluated by qPCR (Figure 5A,B). However, assessment of VM formation using either *siYAP* or *siTAZ* did not yield significant differences in KPC cells. Interestingly, in CT26 cells, while *siTAZ* did not have an effect on VM formation, *siYAP* led to a decrease in the number of junctions and tube length (Supplementary Figure S3A–D). Given these mixed findings, dual knockdown cells were generated, and the expression of their respective genes was evaluated by qPCR and immunoblotting (Figure 5C,D). VM formation was significantly attenuated in dual knockdown cells compared to the control group, as indicated by the number of meshes and junctions and tube lengths (Figure 5E–H). Analysis of known YAP/TAZ transcriptional gene targets by qPCR revealed a cell-dependent loss of expression of several genes, with *Axl* and *Cyr61* significantly downregulated in both cell lines (Figure 5I). Given these findings, we asked whether the expression of YAP/TAZ correlated with the expression of these transcriptional targets in human PDAC and CRC tumors using the Gene Expression Profiling Interactive Analysis (GEPIA) tool [31]. We found a positive correlation between YAP/TAZ expression and their transcription targets in both CRC and PDAC tumors (Supplementary Figure S3E,F).

## 3. Discussion

Abnormal vascular function is a major contributor to tumor invasion and metastasis. Until recently, tumor vascularization was thought to be largely dependent on angiogenesis; instead, current evidence points toward alternative vascularization mechanisms such as tumor co-option of pre-existing vessels and vascular mimicry [4]. This may explain why approved anti-angiogenic therapies only work in a subset of patients, with the majority either developing resistance at the onset or acquiring it in the course of treatment [3]. Moreover, the fact that tumors such as PDAC and metastatic CRC are largely hypovascular points toward alternative vascularization mechanisms supporting their aggressiveness. These findings underscore the importance of investigating alternative vascularization mechanisms and identifying key molecular networks that can serve as therapeutic targets. The majority of VM studies use human cell lines, which is essential in determining heterogenous molecular networks driving vascular mimicry. However, this limits their use in vivo, as most studies rely on subcutaneous xenografts with primary tumor growth as a study outcome. However, our previous studies in melanoma emphasized the importance of the microenvironment in regulating VM formation, given the limitations of subcutaneous models [9,32]. Due to the lack of suitable syngeneic murine models for studying VM, establishing new murine cell lines to investigate this phenomenon is crucial.

Presently, many VM studies using murine cells solely rely on B16-F10, a murine melanoma cell line. This is not surprising, as VM was first discovered in human melanoma [4,5,33], and the majority of studies on VM since its discovery have also been on melanoma. In the current study, we modeled VM formation in vitro in CT26 and KPC cells to understand this non-angiogenic vascularization in mCRC and PDAC, respectively. First, we investigated the triggers of VM formation in these cells. Existing evidence shows that VM formation mimics several steps in angiogenesis, such as ECM remodeling, cell migration, chemotaxis, and tubulogenesis [23]. Like angiogenesis, we found that VM formation is dependent on serum deprivation, which triggers cancer cells to form vessel-like structures when cultured on Matrigel in vitro. However, while angiogenesis is dependent on pro-angiogenic factors, such as VEGFA, secreted in the TME [3], VM formation is independent of these factors, as network formation is not enhanced in the presence of either normoxic or hypoxic tumor-conditioned medium. As hypoxia has been previously reported to elevate VM [25,27], we showed that cells cultured under hypoxia (hypoxia-educated) and used for VM significantly formed more vessel-like structures than their normoxic counterparts. These findings suggest that VM formation is a cell-intrinsic program, which, through specific signaling pathways, instructs cancer cells to generate tube-like structures when cultured on Matrigel.

Previous studies have implicated the Hippo pathway and its effectors, YAP/TAZ, as key regulators of VM formation in solid tumors [9,14,32,34,35]. To investigate the effect of targeting YAP/TAZ on VM formation in vitro, we used Verteporfin and CA3, two well-characterized YAP inhibitors [29]. Verteporfin is a photosensitizer used in the treatment of ocular diseases characterized by abnormal vascularization. It targets Hippo signaling by binding to YAP, which alters its conformation and disrupts its interaction with TEAD [36]. CA3 also inhibits YAP/TAZ activity but is slightly more effective than Verteporfin at lower concentration levels [29], a finding that we also report in this study. In CT26 and KPC cells, CA3 and Verteporfin, at non-cytotoxic levels, significantly inhibited VM formation and decreased tumor chemotaxis. Importantly, these inhibitors suppress YAP/TAZ functions by inhibiting the expression of different target genes that have been implicated in vascular mimicry [2,27]. Moreover, our findings using CA3, especially in CT26 cells, present a dual opportunity for the acute targeting of VM formation and the chronic targeting of proliferation/apoptosis, which ultimately attenuates tumor growth. The differential response to CA3 by these cell lines strongly points toward tumor heterogeneity and has also previously been reported in esophageal adenocarcinoma cells [29]. Accumulation of cells in the sub-G1/G1 phase is usually associated with chemoresistance and evasion of apoptosis, a phenomenon observed in KPC cells post-treatment with CA3 [37,38]. Collectively, these results lay a foundation for the specific targeting of tumor vasculature using non-cytotoxic drug concentrations without necessarily inducing an inflammatory microenvironment.

Pharmacological inhibition of VM was validated by siRNA silencing of YAP/TAZ in both cell lines. Individual knockdown of *YAP* or *TAZ* in KPC cells did not lead to any significant changes in VM formation. On the contrary, in CT26 cells, there was a decrease, in VM formation following individual knockdown of either *YAP* or *TAZ* genes, with significance observed in siYAP knockdown only. These observations are not surprising given the fact that YAP and TAZ, though functionally redundant, have been shown to have non-overlapping roles [39]. A closer analysis of gene expression in either cell line showed a likely compensatory mechanism: when one transcriptional coactivator was silenced, the other remained highly expressed, potentially preserving sufficient activity to maintain VM-supportive transcriptional output. Accordingly, dual silencing of YAP/TAZ led to a significant decrease in VM formation, which corroborated our findings regarding CA3 treatment. In addition, dual knockdown of YAP/TAZ led to decreased expression of downstream transitional targets, including *Axl*, *Ctgf*, *Cyr61*, and *Snail*, which have been demonstrated to promote VM formation [2,35,40,41]. Finally, there was a strong correlation between the expression of YAP/TAZ and their transcriptional targets in PDAC and CRC patients, justifying their clinical significance.

Nonetheless, our work has some limitations. While Matrigel-based assays are the standard for in vitro vascularization assays, they are two-dimensional and do not represent the complex 3D microenvironment of vascularization in vivo [42,43]. In addition, these vascularization assays use monocultures and, therefore, lack the contribution of stromal cells and ECM present in the in vivo microenvironment [43]. This complexity means that it would be difficult to model in vitro to recapitulate the exact mechanism. Despite these limitations, these assays are an important starting point for understanding tumor vascularization. While this work lacks in vivo validation, it is important to reiterate that our aim was to present both cell lines as tools for VM research. Given the lack of data on murine cells undergoing VM, these cells present vital tools to scale VM research in vivo and identify novel players regulating this mechanism. A major weakness of most published VM research is the use of human xenografts in subcutaneous mice to investigate VM in vivo. These models lack different aspects of the TME, which can be countered by using syngeneic orthotopic models. Syngeneic models are disease-relevant and preserve the intricate TME, which makes them valuable for drug discovery studies [17,18]. As VM research is a growing niche, there is limited knowledge about the contribution of stromal cells, the immune system, and ECM in VM development, issues that can be suitably investigated in syngeneic models. For example, orthotopic KPC models, unlike subcutaneous human xenografts, best recapitulate the cellular and stromal features of PDAC’s TME, including its hypovascularity. This implies that investigating VM in an orthotopic pancreas model will provide a more accurate understanding of the molecular events regulating VM, including the contribution of cellular and acellular components of PDAC’s TME. Based on our in vitro findings, efforts are ongoing in the lab to establish stable YAP/TAZ knockout cell lines, followed by VM investigation in syngeneic orthotopic models. Additionally, we will evaluate the effect of anti-angiogenic therapies and YAP/TAZ inhibitors on VM in vivo, either as single agents or in combination therapies. These results will elucidate the underlying molecular mechanisms regulating VM and identify novel drug targets.

In conclusion, we present two established murine cell lines, CT26 and KPC, which can be used to advance further research in VM. As a proof-of-principle, we also provided evidence about the role of YAP/TAZ in VM regulation in vitro. While VM research is still a growing niche, we herein present data showing that VM is an important and targetable mechanism in aggressive tumors such as PDAC and CRC. Given the importance of vascularization in aggressive tumors and metastasis, this work sets a foundation for VM-specific drug discovery in the future. Importantly, given the functional redundancy of YAP/TAZ and the complexity of their signaling network, further investigation of their downstream transcriptional targets and their likely role in VM will also be conducted. Hopefully, this will lead to the development of novel therapies targeting VM in aggressive tumors.

## 4. Materials and Methods

### 4.1. Cell Culture

The 4T1 cell line was a gift from Fred Miller (Wayne State University). CT26 and KPC cells were purchased from the American Type Culture Collection (ATCC). KPC and 4T1 cells were cultured in DMEM GlutaMAX (Cat. No. 31966-021, Thermo Fisher Scientific, Waltham, MA, USA), supplemented with 10% FBS and 1% penicillin–streptomycin. The CT26 cell line was cultured in RPMI 1640 medium (Cat. No. 61870-010, Thermo Fisher Scientific, Waltham, MA, USA), supplemented with 10% FBS (Cat. No. A5256701, Gibco) and 1% penicillin–streptomycin (Cat. No. 15140-122, Thermo Fisher Scientific, Waltham, MA, USA). All cell lines were maintained in a humidified incubator at 37 °C and 5% CO_2_ and routinely tested for mycoplasma infection. For hypoxia exposure, cells were incubated at 37 °C, 5% CO_2_, and 1% O_2_ using the Whitley H35 Hypoxy Station (Don Whitley Scientific, Bingley, UK).

### 4.2. Vascular Mimicry Assay

VM formation in cancer cells was investigated using Matrigel-coated plates in vitro, as previously described [9,32]. Briefly, 50 µL of chilled Matrigel (Cat. No. 354234, Corning Inc., Corning, NY, USA) was added to multiple wells of a cold 96-well plate and incubated for 30 min at 37 °C to allow polymerization. Cancer cells were trypsinized and counted, and 10,000 cells were added to each well. For starvation assays, cells were resuspended in 100 µL of the respective media containing 0%, 2%, and 10% FBS before being added to Matrigel-coated wells.

To assess the effect of tumor-secreted factors on VM formation, conditioned medium from either CT26 or KPC cells was used. Briefly, CT26 or KPC cells were cultured in 10 cm dishes in the respective growth media. At 80% confluence, cells were washed twice with PBS and then sub-cultured in either serum-free RPMI 1640 or DMEM for 24 h under either normoxic or hypoxic conditions. Media were collected, filtered, and subsequently used for VM assays. Tumor cells under normal growth conditions were trypsinized and counted, and 10,000 cells were resuspended in tumor-conditioned medium before seeding on Matrigel-coated wells.

To evaluate the effect of hypoxia on VM formation, cancer cells were sub-cultured in either normoxic or hypoxic conditions for 24 h. Cells were then trypsinized and counted, and 10,000 cells were resuspended in serum-free medium and seeded on Matrigel-coated wells. To investigate the effect of YAP/TAZ inhibition on VM formation, 10,000 cells were resuspended in respective serum-free medium containing 500 nM of CA3 (Cat. No. S8661, MedChemExpress, Monmouth Junction, NJ, USA) or Verteporfin (Cat. No. HY-B0146, MedChemExpress, Monmouth Junction, NJ, USA), followed by seeding on Matrigel-coated wells.

For all assays, VM formation was conducted under standard cell culture conditions (humidified incubator at 37 °C and 5% CO_2_); tube formation was evaluated after 6 h by microscopy; and the number of meshes and junctions and total tube length were evaluated using ImageJ (Version 1.54p), using at least 8 images per independent experiment.

### 4.3. ELISA Assay

Quantitation of VEGFA in cell-conditioned medium was conducted using the Mouse VEGFA ELISA Kit (ELM-VEGF-1, Ray Biotech, Peachtree Corners, GA, USA). Briefly, cells were grown in complete growth medium until 80% confluence. Afterward, the cells were washed three times in PBS and cultured in serum-free medium for 24 h under either normoxic or hypoxic conditions. Conditioned medium was collected and sterile-filtered, and protein concentration was determined using the bicinchoninic acid (BCA) assay, followed by VEGFA quantification according to the manufacturer’s protocol.

### 4.4. Quantitative PCR

Total RNA was isolated using the RNeasy Mini Kit (Cat. No. 74104, QIAGEN, Hilden, Germany), followed by cDNA synthesis using the iScript^TM^ Kit (Cat. No. 1708890, Bio-Rad, Hercules, CA, USA). Quantitative PCR was conducted using the following cycling conditions: 1 cycle at 72 °C for 1 min; 40 cycles of 95 °C for 15 s, 55 °C for 30 s, and 72 °C for 30 s; and 1 cycle at 72 °C for 5 min. The list of primers used can be found in Supplementary Table S1.

### 4.5. Cell Viability Assay

The effect of CA3 and Verteporfin on tumor cell viability was evaluated using the CellTiter-Glo^®^ luminescent cell viability assay (Cat. No. G7572, Promega, Madison, WI, USA) according to the manufacturer’s instructions. Briefly, 5000 cells per well of either cell line were seeded in a 96-well plate and cultured for 24 h in a humidified incubator. Afterward, cells were treated with either 500 nM of CA3 or Verteporfin for 6 h, followed by viability analysis. For IC_50_ determination, cells were treated with different concentrations of either inhibitor for 24 h. Cell viability was then determined using a luminescent assay, and IC_50_ curves were generated using GraphPad Prism Version 10.5.0 (774).

### 4.6. Chemotaxis Assay

Chemotactic migration was assessed using the colorimetric chemotaxis assay (Cat. No. ECM508, Millipore, Burlington, MA, USA). Before the assay, tumor cells were starved for 24 h. Cells were then dissociated and counted, and 1 × 10^5^ cells were resuspended in serum-free medium containing either 500 nM of CA3 or Verteporfin and seeded into a transwell insert. Then, 10% FBS-medium was used as a chemoattractant. After 14 h, the migrated cells were stained and imaged, and chemotaxis was quantified by manually counting the migrated cells.

### 4.7. Colony Formation Assay

Briefly, KPC cells were seeded at a low density (250 cells per well) in 6-well plates and cultured overnight to allow for cell settling. The following day, the medium was discarded and replaced with fresh growth medium containing either 500 nM of CA3 or Verteporfin. DMSO was used as a treatment control. Medium changes were performed after 3 days, and colony formation was evaluated after 6 days. Established colonies were washed with PBS and incubated for 10 min in cell stain (Cat. No. 90144-200KL, Millipore, Burlington, MA, USA). The dye was discarded, and colony formation was evaluated via microscopy and manual counting.

### 4.8. Cell Proliferation Assay

Cell proliferation was evaluated using the IncuCyte X^TM^ Live Cell Imager. Briefly, 10,000 cells were seeded in each well of a 96-well plate and left to settle overnight in a humidified incubator. The growth medium was replaced using fresh complete growth medium containing 500 nM of Verteporfin, 500 nM of CA3, and the respective CA3 IC_50_ values for CT26 and KPC. DMSO was used as a treatment control. The plate was placed inside the IncuCyte X^TM^ Live Cell Imager and growth-monitored for 48 h, with live imaging of proliferating cells taken every 4 h. Proliferation differences between the two groups were determined using the area under the curve (AUC) method.

### 4.9. Apoptosis Assay

Cancer cell apoptosis was evaluated with Annexin V/7AAD double staining and quantified using flow cytometry. Briefly, cells were seeded in 10 cm dishes and sub-cultured for 24 h. This was followed by treatment with CA3 (IC_50_ values for respective cell lines) for 24 h, with DMSO as a control. Cells were harvested, washed in PBS, and stained with Annexin V (Cat. No. A13201, Life Technologies, Waltham, MA, USA) and 7AAD (Cat. No. 51-68981E, BD Pharmingen, Franklin Lakes, NJ, USA). Stained cells were analyzed using the BD FACSCelesta^TM^ Flow Cytometer (BD Biosciences, San Jose, CA, USA).

### 4.10. Cell Cycle Analysis

Cells were treated with CA3 (IC_50_ values) for 24 h, harvested, and fixed in 70% ethanol at 4 °C overnight. After fixation, cells were washed twice with PBS and incubated with 100 μg/mL of RNase A (Cat. No. 12091-021, Invitrogen, Waltham, MA, USA) and 50 μg/mL of propidium iodide (Cat. No. 421301, BioLegend, San Diego, CA, USA) for 30 min in the dark at room temperature. DNA content was analyzed using flow cytometry, and the distribution of cells in the Sub-G1, G1, S, and G2 phases was quantified using the FlowJo software (version 10.10.0).

### 4.11. Western Blot Analysis

Total protein was extracted from growing cells using RIPA lysis buffer containing EDTA, phosphatase, and protease inhibitors. Protein concentration was determined using the BCA assay. An equal amount of protein samples were denatured at 70 °C for 10 min with 4x Laemmli Buffer, separated using 4–12% Bis-Tris gels (Cat. No. NP0335BOX, Thermo Fisher Scientific, Waltham, MA, USA) under reducing conditions, and transferred to PVDF membranes (Cat. No. IPVH00010, Millipore, Burlington, MA, USA). Following blocking with 5% skim milk in TBS-T for 1 h at room temperature, membranes were incubated overnight at 4 °C with the following primary antibodies: YAP/TAZ (Cat. No. 8418), YAP (Cat. No. 14074), TAZ (Cat. No. 83669) all from Cell Signaling Technology (Danvers, MA, USA), and β-Actin (Cat. No. sc-47778, Santa Cruz Biotechnology, Dallas, TX, USA). After washing, membranes were incubated with HRP-conjugated anti-mouse (Cat. No. 31430) or anti-rabbit (Cat. No. 31460) both from Thermo Fisher Scientific (Waltham, MA, USA) secondary antibodies for 1 h at room temperature. Membranes were developed using chemiluminescent reagents and imaged using the ChemiDoc^TM^ imaging system (BioRad, Hercules, CA, USA).

### 4.12. siRNA Silencing

CT26 and KPC were cultured overnight and transfected with 10 nM of targeted siRNAs against YAP (5′-CCACCAAGCUAGAUAAAGAAAGCTT-3′), against TAZ (5′-GAUACUUCCUUAAUCACAUAGAGAA-3′), or with a scrambled siRNA as a control, all introduced by Lipofectamine RNAiMax transfection reagent (Cat. No. 13778150, Invitrogen, Waltham, MA, USA) in reduced serum-free media (Opti-MEM I (1X), Cat No. 31985-070, Gibco, Waltham, MA, USA). After 24 h, the transfection mixture was replaced with complete growth medium, and the transfected cells were cultured for a further 48 h. Afterward, transfected cells were harvested and used for VM and chemotaxis assays, as previously described. YAP/TAZ knockdown was confirmed via quantitative PCR (qPCR) and immunoblotting.

### 4.13. Statistical Analysis

Statistical analysis was performed using GraphPad Prism version 10.4.1 (627), with unpaired two-tailed *t*-test with Welch’s correction to compare two experimental groups and Welch’s ANOVA test paired with Dunnett’s T3 multiple comparison for more than two groups. For all in vitro experiments, three independent experiments were used as the sample size. All quantitative data are presented as mean ± SEM unless otherwise stated, and *p* < 0.05 is considered statistically significant.

## Data Availability

The original contributions presented in this study are included in the article/supplementary material. Further inquiries can be directed to the corresponding author(s).

## Supplementary Materials

The following supporting information can be downloaded at https://www.mdpi.com/article/10.3390/ijms26189129/s1.
