# Supplementary material for: Murine Cell Line Models for Vascular Mimicry: The Role of YAP/TAZ Signaling
Source: Int J Mol Sci. 2025 Sep 18;26(18):9129. doi: 10.3390/ijms26189129 (PMC12471087; doi:10.3390/ijms26189129)
Supplement: Supplementary file 1 [file ijms-26-09129-s001.zip › ijms-3805567-figure captions.pdf]

## Supplementary Figure Legends

**Supplementary Figure S1:** Images of 4T1 cells undergoing VM formation in the presence of (**A**) increasing FBS concentrations and (**B**) tumor-conditioned medium. (**C**) Images of CT26 and KPC cells VM formation in the presence of normoxic or hypoxic tumor-conditioned medium. Quantification of the number of (**D**) meshes, (**E**) junctions and (**F**) tube length in CT26 and KPC cells undergoing VM in the presence of normoxic and hypoxic tumor-conditioned medium (n = 8 per independent biological experiment). All data are expressed as mean ± SEM of three independent experiments and statistical significance is determined using the ordinary Welch's ANOVA test paired with Dunnett's T3 multiple comparisons test.

**Supplementary Figure S2:** (**A**) CT26 and KPC cell viability following treatment with either 500 nM CA3 or Verteporfin for 6 hours. (**B**) Gene expression analysis of YAP/TAZ transcriptional targets *Birc5*, *Twist1*, and *Areg* in CT26 and KPC cells following treatment with either CA3 or Verteporfin (**C - D**) Cell proliferation of CT26 and KPC following treatment with VM-inhibiting concentration of CA3 and Verteporfin for 72 hours. (**E - F**) Colony formation of KPC cells in the presence of VM-inhibiting concentrations of CA3 and Verteporfin. The number of colonies were counted manually and averaged from three separate experiments. (**G**) Images of chemotactic CT26 and KPC cells. (**H**) Graph showing the relative chemotactic migration of CT26 and KPC cells in the presence of YAP/TAZ inhibitors quantified by manual counting. All data are expressed as mean ± SEM of three independent experiments and statistical significance is determined using Welch's ANOVA test paired with Dunnett's T3 multiple comparisons test: *$p < 0.05$ and **$p < 0.01$. (**I - J**) $IC_{50}$ determination of CA3 and Verteporfin in CT26 and KPC cells after 24 hours using the Cell Titer Glo Luminescent Assay. Error bars denote the standard deviation of three independent replicates for each tested dose.

**Supplementary Figure S3:** (**A**) Images of CT26 and KPC cells undergoing VM formation following individual knockdown of either *YAP* or *TAZ* genes. Quantification of the number of (**B**) meshes, (**C**) junctions and (**D**) tube length in CT26 and KPC cells undergoing VM following individual knockdown of either *YAP* or *TAZ* genes (n = 8 per independent experiment). All data are expressed as mean ± SEM of two independent experiments and statistical significance is determined using the Welch's ANOVA test paired with Dunnett's T3 multiple comparisons test: *$p < 0.05$ and ***$p < 0.001$. The public dataset GEPIA showed a positive correlation between *YAP/TAZ* expression (*TAZ* herein written as *WWTR1*) and their transcriptional targets in (**E**) PDAC and (**F**) CRC patients.
