# Supplementary figures and images for: Murine Cell Line Models for Vascular Mimicry: The Role of YAP/TAZ Signaling
Source: Int J Mol Sci. 2025 Sep 18;26(18):9129. doi: 10.3390/ijms26189129 (PMC12471087; doi:10.3390/ijms26189129)

# Supplementary Figure S1

**A**

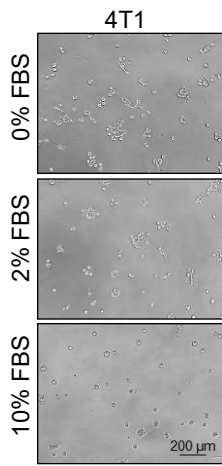

**B**

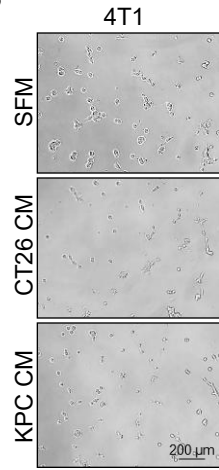

**C**

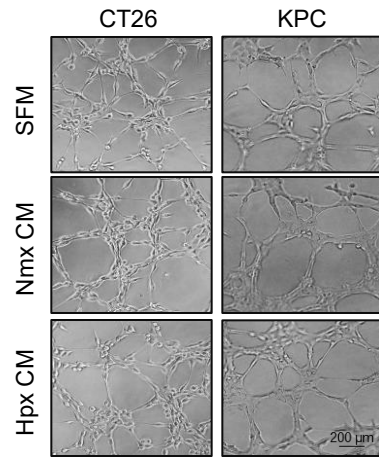

**D**

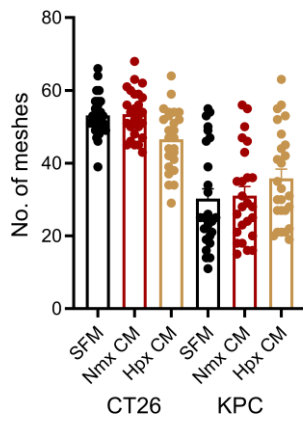

**E**

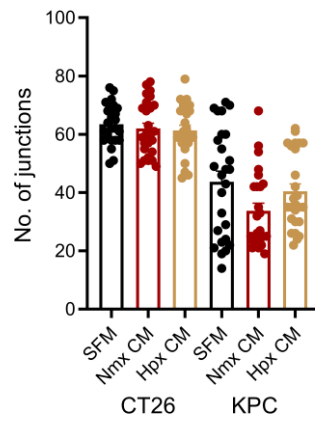

**F**

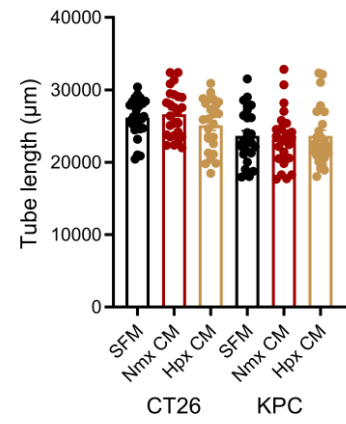

# Supplementary Figure S2

**A**

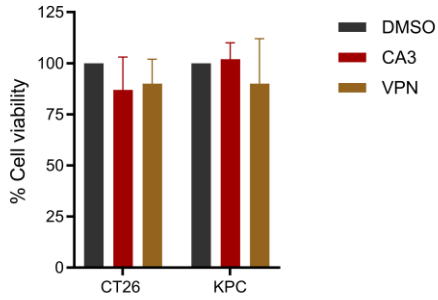

**B**

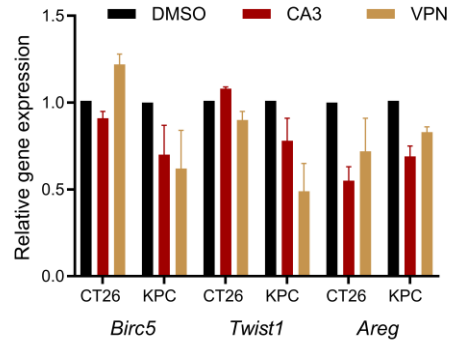

**C**

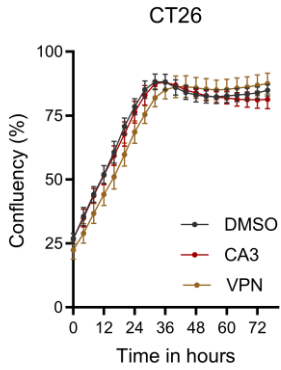

**D**

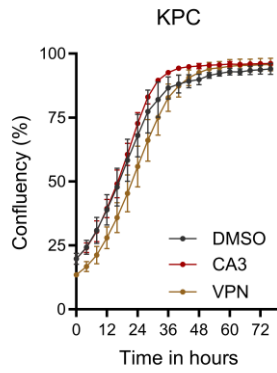

**E**

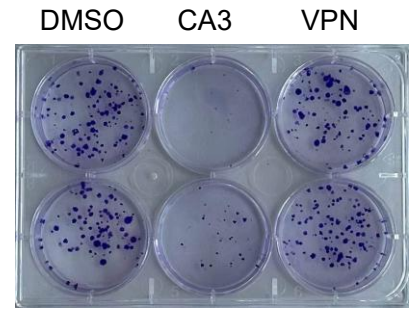

**F**

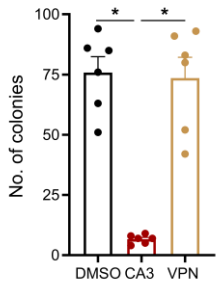

**G**

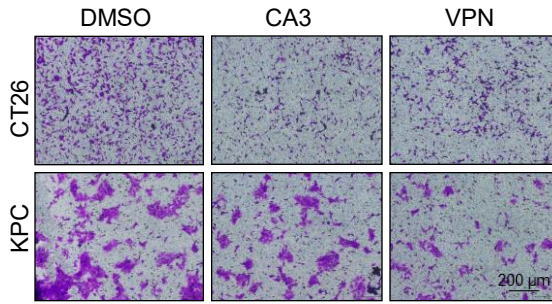

**H**

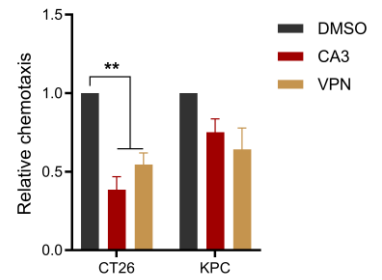

**I**

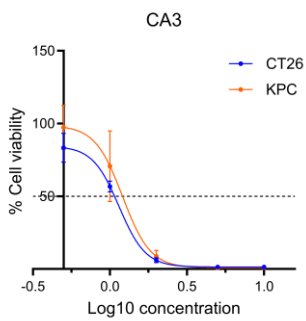

**J**

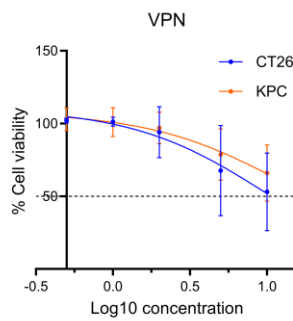

Supplementary Figure S3

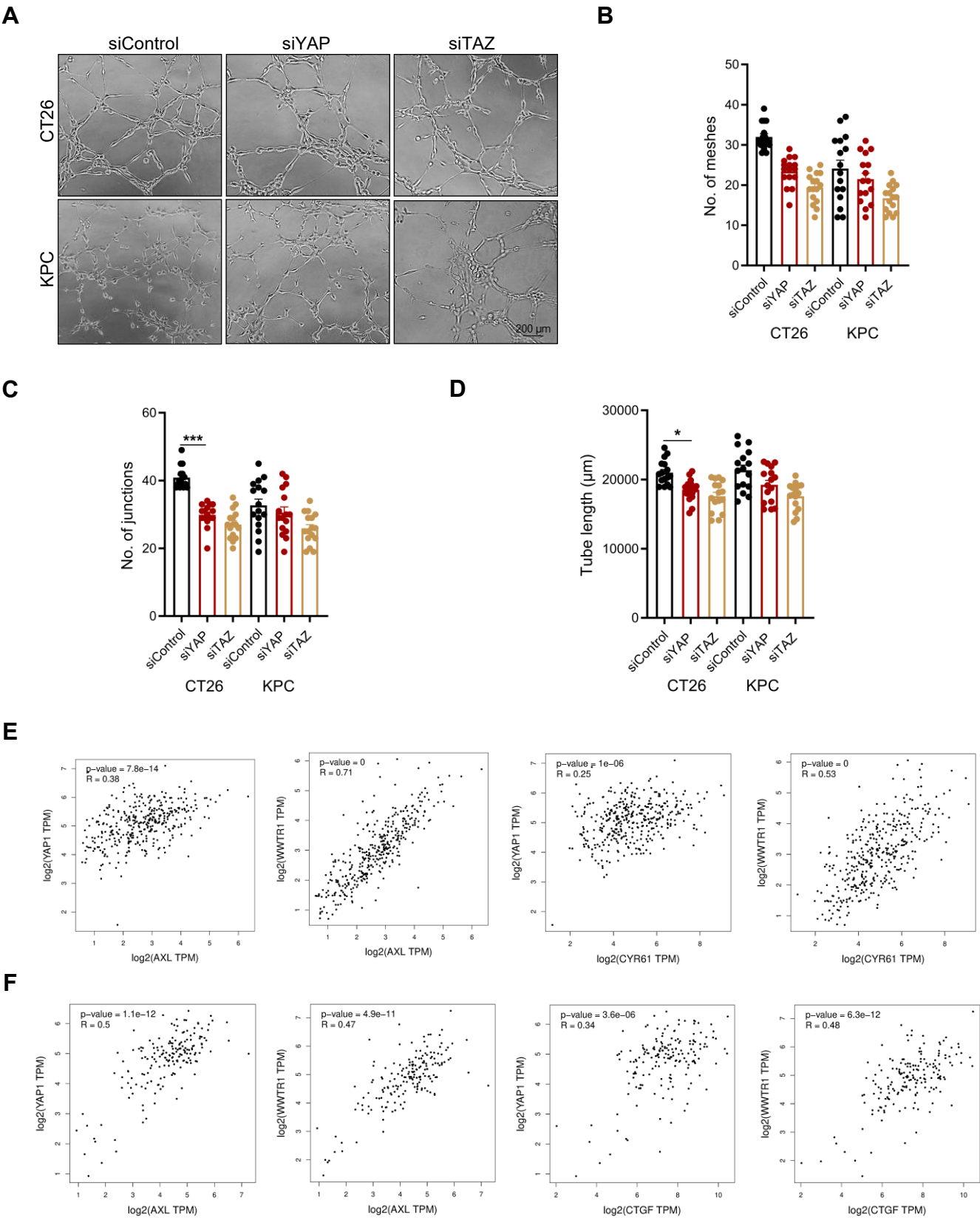

Supplement: Supplementary file 1 [file ijms-26-09129-s001.zip › ijms-3805567-figure images.pdf]
